# Two-dimensional penta-Sn_3_H_2_ monolayer for nanoelectronics and photocatalytic water splitting: a first-principles study

**DOI:** 10.1039/c8ra00320c

**Published:** 2018-03-27

**Authors:** Peng Zhang, Xibin Yang, Wei Wu, Lifen Tian, Daxi Xiong, Heping Cui, Xianping Chen, Kai Zheng, Huaiyu Ye

**Affiliations:** State Key Laboratory of Advanced Power Transmission Technology Beijing 102209 China; Suzhou Institute of Biomedical Engineering, Chinese Academy of Sciences Suzhou 215163 China; Key Laboratory of Optoelectronic Technology & Systems, Education Ministry of China, Chongqing University and College of Optoelectronic Engineering, Chongqing University Chongqing 400044 China kaizheng@cqu.edu.cn huaiyuye@cqu.edu.cn; School of Mechanical and Electrical Engineering, Guilin University of Electronic Technology Guilin 541004 China

## Abstract

Exploring two-dimensional materials with novel properties is becoming particularly important due to their potential applications in future electronics and optoelectronics. In the current work, the electronic and optical properties of penta-Sn_3_H_2_ are investigated by density-functional theory. By assessing the phonon spectrum, we find that penta-Sn_3_H_2_ monolayer is energetically more favorable compared with pristine penta-stanene due to hydrogenation transforming the sp^2^–sp^3^ hybrid orbitals into sp^3^ hybridization. Our calculations revealed that penta-Sn_3_H_2_ is a semiconductor with indirect band gaps of 1.48 eV according to the GGA functional (2.44 eV according to the HSE06 functional). Moreover, the electronic structures of penta-Sn_3_H_2_ can be effectively modulated by biaxial tensile strain. Meanwhile, our calculations reveal that the indirect to direct band gap transition can be achieved in this monolayer sheet by >4% biaxial strain. On the other hand, the well-located band edge and visible light absorption make penta-Sn_3_H_2_ a potentially promising optoelectronic material for photocatalytic water splitting.

## Abstract 2

Exploring two-dimensional materials with novel properties is becoming particularly important due to their potential applications in future electronics and optoelectronics.

## Graphical abstract

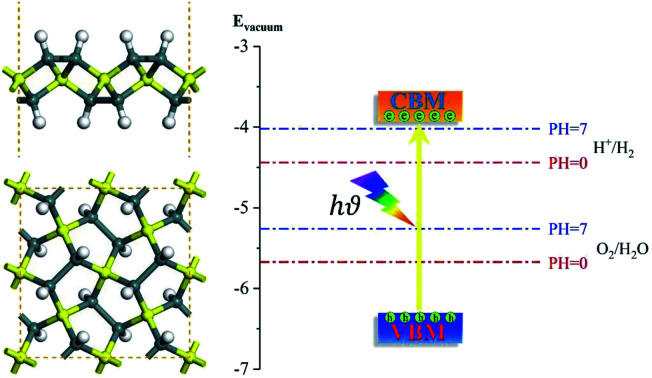

## 1. Introduction

Ultrathin two-dimensional (2D) nanomaterials have attracted a remarkable increase in interest since the successful fabrication of graphene in 2004.^1–3^ Graphene is a material with ultrahigh mobilities, owing to its extremely low carrier effective mass, and hence it is viewed as a promising candidate for high-speed electronic devices.^4,5^ Inspired by the unique properties originating from 2D nanostructures, group-IV (silicon, germanium, and tin) analogues have been theoretically and experimentally investigated.^6,7^ However, pristine graphene, silicene, germanene and stanene have a common absence of band gap, which is deleterious for their applications in modern electronics.^8–10^ Very recently, a pure pentagonal structure has been proposed for graphene, silicene and germanene, which achieves Cairo pentagonal tiling in the sheet.^11–13^ These novel 2D group-IV allotropes, named penta-graphene, penta-silicene and penta-germanene, respectively, presenting a tetragonal lattice with threefold and fourfold coordinated hybrid atom sheets, was proposed by computational modeling. First-principles calculations have predicted that penta-graphene is stable at room temperature with a quasi-direct band gap of 3.25 eV.^11^ While penta-silicene and penta-germanene are not stable at room temperature.^12,13^

Chemical functionalization is an efficient approach for modulating the structural, electronic, and magnetic properties of 2D nanomaterials, and, in particular, hydrogenation is one of the most effective ways.^14–17^ Previous studies have shown that the energetic and mechanical stabilities of penta-silicene and penta-germanene can be greatly improved *via* functionalization (hydrogenation or fluorination). The electronic and mechanical properties can also be effectively modulated, and an unexpected enhancement in thermal conductivity can be obtained for hydrogenated penta-graphene. Here, a natural question comes to mind: is penta-stanene a stable structure? Would those interesting properties and changes occur in the structural, electronic, and optical properties of monolayer penta-stanene when it is functionalized?

Here, we focus on an exploration of penta-stanene, penta-Sn_3_H_2_ and penta-Sn_3_F_2_ as nano-electronic materials and photocatalysts by a first-principles method. We first determine the stability of the materials, followed by a study of the electronic structures and the bonding of atoms of penta-Sn_3_H_2_ using a GGA-PBE functional and an accurate hybrid density functional. Next, we study how mechanical strains can be used to tune the band structures. And then we compute the properties related to photocatalytic water splitting, such as band edge positions and optical absorption. Finally, we address the surface morphology of penta-Sn_3_H_2_ by calculating the scanning tunneling microscope images.

## 2. Computational methods

The calculations are based on spin-polarized density functional theory with the Perdew–Burke–Ernzerhof (PBE) form of the generalized gradient approximation (GGA)^18^ to the exchange–correlation potential, as implemented in the Cambridge Sequential Total Energy Package (CASTEP) code.^19^ The kinetic energy cutoff of the plane wave is set to be 500 eV on a Monkhorst–Pack grid using an adequate *k*-point scheme of 9 × 9 × 1 for geometrical optimization and 20 × 20 × 1 *k*-points for band structure calculations, which ensures that the configurations fully converge. In addition, the geometry of the configuration is optimized by using a Broyden–Fletcher–Goldfarb–Shanno (BFGS) minimizer. The self-consistent convergence accuracy is set to be 1 × 10^−6^ eV per atom, the convergence criterion for the force between atoms is 0.03 eV per Å, and the maximum displacement is 1.0 × 10^−3^ Å. A sufficiently large 20 Å vacuum region is used to separate the two-dimensional structures to rule out any interaction among the neighboring layers along the c-axis. Grimme's DFT-D dispersion correction is applied to account for the long-range van der Waals interactions.^20^ It is well known that GGA always underestimates the band gap value of 2D systems. So, we use the hybrid (HSE06) functional^21,22^ to verify and confirm the band structure of monolayer penta-Sn_3_H_2_.

According to Mulliken's electronegativity theory, the conduction band potential can be calculated using the equation:

1 *E*_VB_ = *χ* − *E*^C^ + 0.5*E*_g_

2 *E*_CB_ = *E*_VB_ − *E*_g_

where *χ* is the absolute electronegativity of the semiconductor, *E*^C^ is the energy of the free electron on the hydrogen scale (∼4.50 eV), and *E*_g_ is the band gap of the semiconductor.

The optical properties were calculated with a plane-wave kinetic energy cutoff of 500 eV, and the *k*-point mesh was set to 20 × 20 × 1. To calculate the optical properties of penta-Sn_3_H_2_, the absorption coefficient can be obtained from the following equation:^23^3
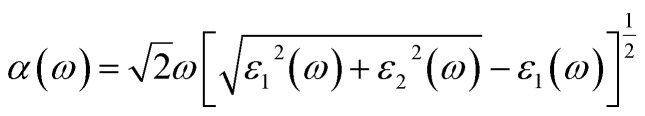
where the *ε*_1_ and *ε*_2_ are the real and imaginary dielectric functions, respectively.

## 3. Results and discussion

### 3.1 Crystal structures and stability

**Fig. 1:**
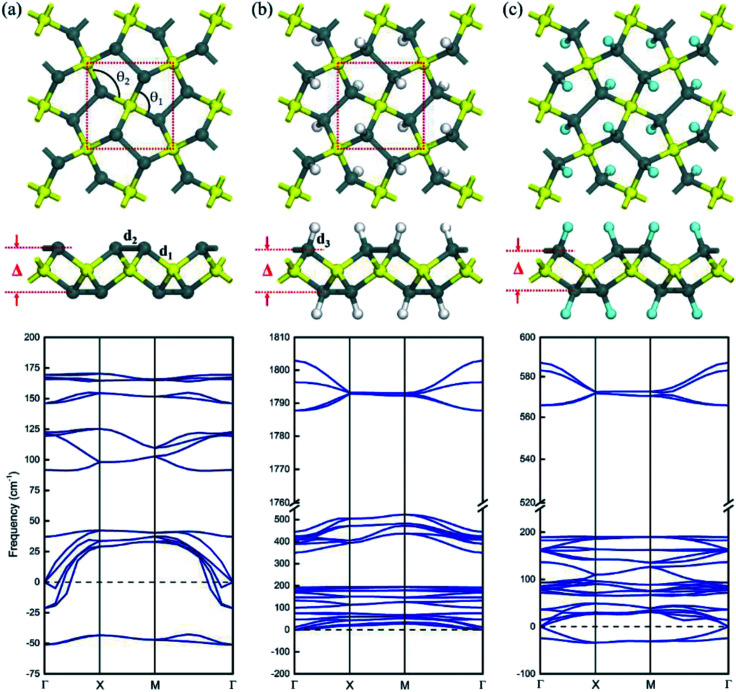
Top and side views of the optimized structures along with their phonon dispersions of (a) pristine (pSn) and (b) hydrogenated (H–pSn–H) and (c) fluorinated (F–pSn–F) pSns. The squares marked by the red dashed lines denote the unit cells. The highlighted yellow spheres refer to the fourfold coordinated Sn1 atoms in each system.

The atomic structural geometry of pristine pSn is shown in Fig. 1a, presenting a tetragonal lattice consisting of four pentagons in the primitive cell. The optimized lattice constant of the pristine pSn is *a* = 6.30 Å, and the structure possesses *P*4̄21*m* symmetry (space group no. 113). The unit cell comprises two tetracoordinated Sn atoms and four tricoordinated Sn atoms, and, for convenience, they are labeled Sn1 and Sn2, respectively. For the pSn nanostructure, the bond length of Sn1–Sn2 atoms is *R*_1_ = 2.85 Å and that of Sn2–Sn2 atoms is *R*_2_ = 2.91 Å. The basal plane of pSn is puckered, and the buckling distance (determined by the vertical height difference between the Sn1 and Sn2 layers shown in Fig. 1a) is *Δ* = 3.19 Å, which is much larger than the corresponding values for penta-graphene, penta-silicene or penta-germanene. This is attributed to the larger atomic radii of tin atoms. Moreover, the bond angle of Sn2–Sn1–Sn2 is *θ*_1_ = 111.8°, and the bond angle of Sn1–Sn2–Sn1 is *θ*_2_ = 102.9°, indicating distinct sp^3^ hybridized bonds. To verify whether penta-Sn remains stable under displacement of the constituent atoms, we carry out calculations on the phonon frequency spectrum. It is well known that if the vibration frequency of specific modes is imaginary, the corresponding systems would be dynamically unstable. We have measured the phonon spectrum of the pentagonal structure, as shown at the bottom of Fig. 1a. The presence of an imaginary vibration which is below 0 in the phonon dispersion shows that the pSn structure is dynamically unstable. The detailed analysis shows that the two imaginary vibrations around the *Γ*-point can be ascribed to the tricoordinated Sn2 atoms. Based on valence-bond theory, we found that the Sn2 atoms are only bonded to three neighboring Sn atoms but Sn1 atoms bond to four neighboring Sn atoms, so Sn2 atoms prefer to transform into sp^2^ hybridization, causing soft modes to collapse the 2D sheet. Overall, the Sn2 atoms tend to distort the pentagonal structure, leading to dynamic instability.

To improve the structural stability of pSn, the key is to stabilize the buckled structure of Sn2 atoms. Thus, a powerful route to enhance the stability is chemical functionalization, which has been widely used to tune the properties of low-dimensional nanostructures,^24^ including Sn-based nanomaterials.^25–28^ Indeed, previous experimental studies have shown that both hydrogenated- and fluorinated-stanene sheets with graphene-like lattices are stable at room temperature.^25,29^ Therefore, we explore the structural stability of hydrogenated and fluorinated penta-stanene. Considering that there are four unsaturated Sn2 atoms per unit cell, we decorate the top of the Sn2 atoms by hydrogenation and fluorination, where all the Sn atoms become tetracoordinated, as shown in Fig. 1b and c. They are labeled H–pSn–H and F–pSn–F, respectively, and the formed monolayers are named penta-Sn_3_H_2_ and penta-Sn_3_F_2_. The structural parameters of penta-Sn_3_H_2_ are listed in Table 1. It can clearly be seen that the hydrogenation in H–pSn–H results in a decrease in the bond length, which is due to a significant reduction in the lattice constant. It can be noticed that the buckling distances of the penta-Sn_3_H_2_'s have experienced little change compared with those of pristine penta-stanene. To examine the dynamic stability of the hydrogenated and fluorinated nanostructures, we performed phonon calculations of H–pSn–H and F–pSn–F sheets, as shown at the top of Fig. 1b and c. Obviously, there are no imaginary vibrations throughout the whole Brillouin zone (BZ), confirming the dynamic stability of monolayer penta-Sn_3_H_2_. But in a fluorinated pSn sheet, the imaginary vibration in the phonon dispersion manifests that this structure is dynamically unstable.

**Table 1:** Optimized structure parameters, distance *Δ* between the top and bottom layers of the tin atoms, electronic band gap *E*_g_ calculated by using the PBE/HSE06 functional, and binding energy *E*_b_ of penta-Sn_3_H_2_. Symbol illustration: *d*_1_ (Sn1–Sn2 bond length in Å), *d*_2_ (Sn2–Sn2 bond length in Å), *d*_3_ (Sn–H bond length in Å), *θ*_1_/*θ*_2_ (Sn2–Sn1–Sn2/Sn1–Sn2–Sn1 angle in deg), *Δ* (buckling distance in Å), *a* (lattice constant in Å), *E*_g_-PBE/*E*_g_-HSE06 (in eV), *E*_b_ (binding energy in eV/per atom)

Conformation	*d*_1_ (Å)	*d*_2_ (Å)	*d*_3_ (Å)	*θ*_1_/*θ*_2_ (deg)	*Δ* (Å)	*a* (Å)	*E*_g_-PBE	*E*_g_-HSE06	*E*_b_	References
H–pSn–H	2.81	2.82	1.75	107.2/104.1	3.06	6.27	1.48	2.44	3.52	This work
H–h-Sn–H	—	2.82	1.73	—	0.94	4.65	0.24	1.22	—	Ref. 23
H–pGe–H	2.48	2.47	1.56	106.3/105.2	2.64	5.57	1.92	2.60	0.52	Ref. 12
H–pSi–H	2.37	2.37	—	—	2.52	—	1.74	2.46	—	Ref. 11
H–pG–H	1.55	1.55	1.10	116.9/105.9	1.62	—	4.29	5.35	3.65	Ref. 24

**Fig. 2:**
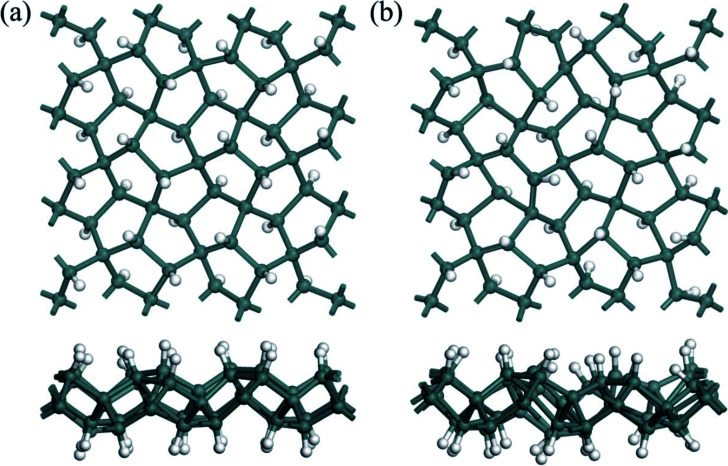
Top and side views of snapshots of penta-Sn_3_H_2_ monolayer equilibrium structures at (a) 300 K and (b) 900 K at the end of 5 ps first-principles molecular dynamics simulations.

To address the enhancement in the thermal stability induced by hydrogenation, *ab initio* molecular dynamic (AIMD) simulations with a GGA-PBE functional were performed by using DMol^3^ code at 300 and 900 K, respectively. The 2D chemically hydrogenated sheet is expanded to a 3 × 3 supercell consisting of 90 atoms. The penta-Sn_3_H_2_ sheets are found to sustain their integrated nanostructures during the AIMD simulations at 300 and 900 K. Snapshots of geometric structures at the end of the AIMD simulations for the monolayer penta-Sn_3_H_2_ are presented in Fig. 2, which shows that the atomic configuration of the monolayers remains nearly intact after heating for 5 ps, indicating that the monolayers are thermally stable even at relatively high temperature. Thus, H–pSn–H is not only dynamically stable but also thermally stable at room temperature and even at a temperature of 900 K. Next, we will focus on the electronic structures of monolayer penta-Sn_3_H_2_, and the electronic properties of pristine penta-Sn, but fluorination will not be considered because of their instability.

### 3.2 Electronic structures

**Fig. 3:**
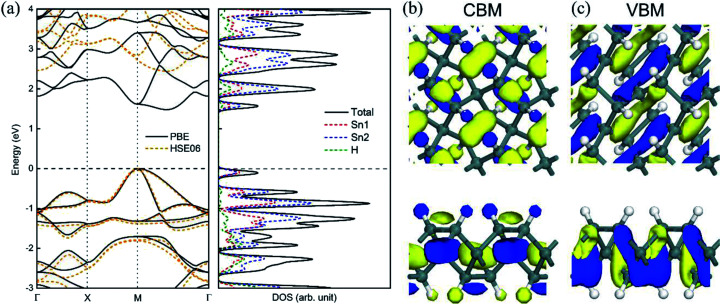
(a) Electronic band structure and atom-decomposed partial DOS of monolayer penta-Sn_3_H_2_. Black solid lines and orange dashed lines correspond to the PBE and HSE06 results, respectively. (b and c) Isosurface plots of the corresponding band-decomposed charge density distributions corresponding to CBM and VBM are displayed on the right-hand side of the PDOS result. The isovalue is 0.04 e Å^−3^. The Fermi level is set to zero and indicated by the black dashed line.

The calculated electronic structures and the corresponding partial density of states (PDOSs) of penta-Sn_3_H_2_ are shown in Fig. 3a. Clearly, penta-Sn_3_H_2_ possesses an indirect band gap of 1.48 eV at the PBE level. Its valence bond maximum (VBM) is located at the *M*-point, whereas its conducting bond minimum (CBM) is located about halfway along the *Γ*–*M* path in the first Brillouin zone. This feature is the same as for hydrogenated penta-germanene, but different from that of hydrogenated penta-silicene or hydrogenated penta-graphene, whose CBMs are located at the *Γ*-point. Considering the underestimation of the PBE functional in predicting electronic band gaps of semiconductors, the hybrid HSE06 functional was adopted here to gain a more accurate description of the band structure of penta-Sn_3_H_2._ The band structure obtained by using the HSE06 functional is similar to that obtained by PBE functional, but the band gap changes to 2.44 eV, suggesting that hydrogenation effectively modulates the electronic structure of penta-stanene and enlarges its band gap to a more favorable range for electronic applications. In addition, the band gaps of stanene from previous studies are presented in Table 1 for comparison. Furthermore, the atom-decomposed partial DOSs were calculated for analysis of the electronic band structures. The partial DOS of penta-Sn_3_H_2_ shows that the electronic states near the Fermi level are mainly contributed by Sn1 and Sn2 atoms. To shed light on the electronic distribution in monolayer penta-Sn_3_H_2_, the charge density distribution was computed and is illustrated in Fig. 3b. The VBM of penta-Sn_3_H_2_ is mainly contributed by the Sn2–Sn1 bonding state and partially by the Sn2–Sn2 bonding state, indicating an *σ*_pp_ character. In contrast, its CBM (in Fig. 3c) is composed of the Sn2–H antibonding state, indicating an 
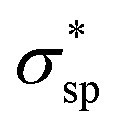
 character.

**Fig. 4:**
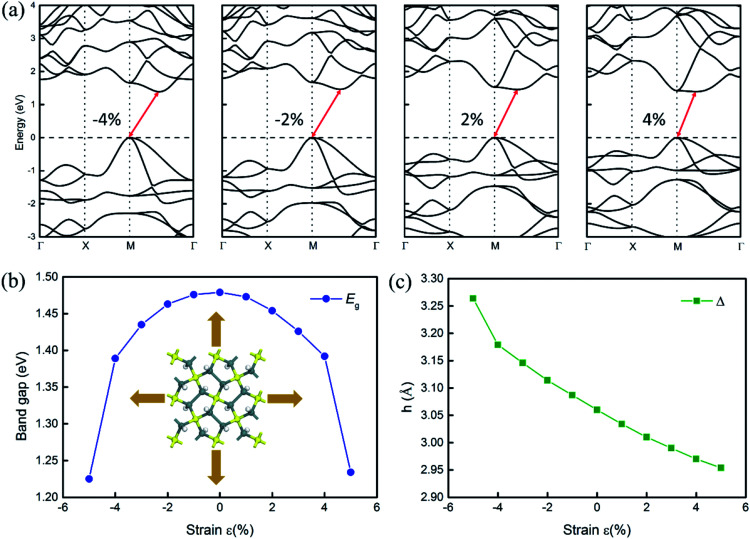
Effects of tensile strain on the electronic structures of penta-Sn_3_H_2_. The electronic band structures of (a) penta-Sn_3_H_2_ under different tensile strains. The band structures shown here are calculated by the PBE functional. (b) Biaxial strain dependence of the band gap (*E*_g_) of penta-Sn_3_H_2_. (c) The variation in buckling distance as a function of biaxial strain for penta-Sn_3_H_2_, confirming that the unstrained nanostructure corresponds to the lowest total energy. Band structures based on the PBE functional under certain external tensile strains are listed in the figures.

An external strain has been widely used to tune the electronic structure and thermal properties of materials.^30^ Thus, we investigate the effects of biaxial strain on the electronic structures of penta-Sn_3_H_2_ and hope that the indirect–direct band gap transition can be obtained for H–pSn–H by tensile strain. In this study, the biaxial strain is simulated by varying the in-plane lattice to a series of values, which are larger/smaller than those of the equilibrium structure. The strain imposed on the structure is defined as *ε* = (*a* − *a*_0_)/*a*_0_, where *a*_0_ and *a* denote the lattice constants of the unstrained and strained systems, respectively. Considering the differences in the electronic structures obtained by PBE and HSE06, we calculate the electronic structures at PBE level, as shown in Fig. 4. Correspondingly, the HSE06 band gap values can be roughly estimated by a scissor operation.

As explicitly shown in Fig. 4a, a quasi-direct band gap at the *M* point can be obtained for the 2D penta-Sn_3_H_2_ sheet when *ε* = 4% biaxial strain is applied. It is obvious that the direct band gap can be achieved when the biaxial strain is continually enlarged. The presence of the indirect–direct semiconductor transition is favorable for its potential applications, and this phenomenon can be understood from the charge transfer between Sn2 and Sn1 atoms. In detail, once a tensile stretch has been applied to the nanostructure, the Sn1–Sn2 bond length is increased from 2.812 to 2.874 Å when *ε* = 4% and the buckling distance (*Δ*) is also monotonously decreased. As the tensile stretch increased monotonously, the Sn1 atom's induced energy level with the 
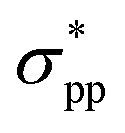
 character is shifted downward at the *M*-point and the Sn2 atom's induced energy level (at the halfway point of *M*–*Γ* path) with the 
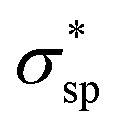
 character is shifted upward. Once the critical strain of *ε* = 4% is achieved, the 
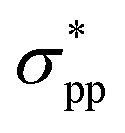
-characterized state eventually becomes CBM to replace the original 
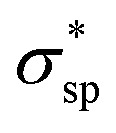
 state, leading to an indirect–direct transition for penta-Sn_3_H_2_. Generally, the band gap of penta-Sn_3_H_2_ decreases in a linear way with a continuous increase in stretch strain. Conversely, when a compression tensile strain is applied, the 
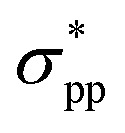
-characterized level is shifted upward and the 
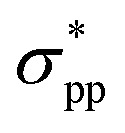
-characterized one is shifted downward. As a consequence, the indirect band gap is reduced as a compression strain is exerted on the penta-Sn_3_H_2_ nanosheet, as shown in Fig. 4b. For the 2D penta-Sn_3_H_2_ nanosheet, it is interesting to find that not only can its band gap be tuned over a wide range of 1.225–1.479 eV (2.182–2.436 eV at HSE06 level according to the scissor operation), but the indirect to direct band gap transition can be also obtained for this semiconductor. Most importantly, the strain range calculated in our study is generally achievable for experimental realization.^31^ For instance, a 2D MoS_2_ nanomaterial can be subjected to an external strain of 11%.^32^

### 3.3 Photocatalytic properties

To generate hydrogen and oxygen from water splitting, the band edges should be positioned appropriately with respect to the redox levels of water. The band structures with an HSE06 function reveal that the band gap of a penta-Sn_3_H_2_ monolayer is 2.44 eV, which exceeds the free energy of water splitting of 1.23 eV. In order to meet the conditions for splitting water, the band edges must range over the redox potentials of water: that is, its conduction band minimum (CBM) energy must be higher than the reduction potential of H^+^/H_2_ and its valence band maximum (VBM) energy must be lower than the oxidation potential of O_2_/H_2_O. For the water splitting reaction, the redox potential depends on the pH value. The standard reduction potential for H^+^/H_2_ was calculated from 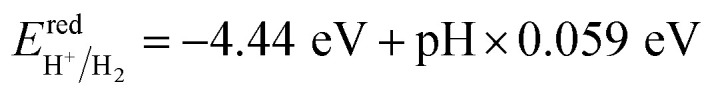 and the oxidation potential for O_2_/H_2_O was calculated from 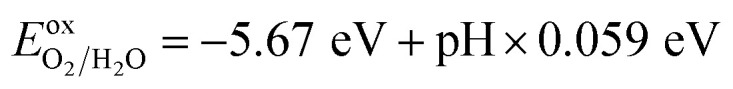.

**Fig. 5:**
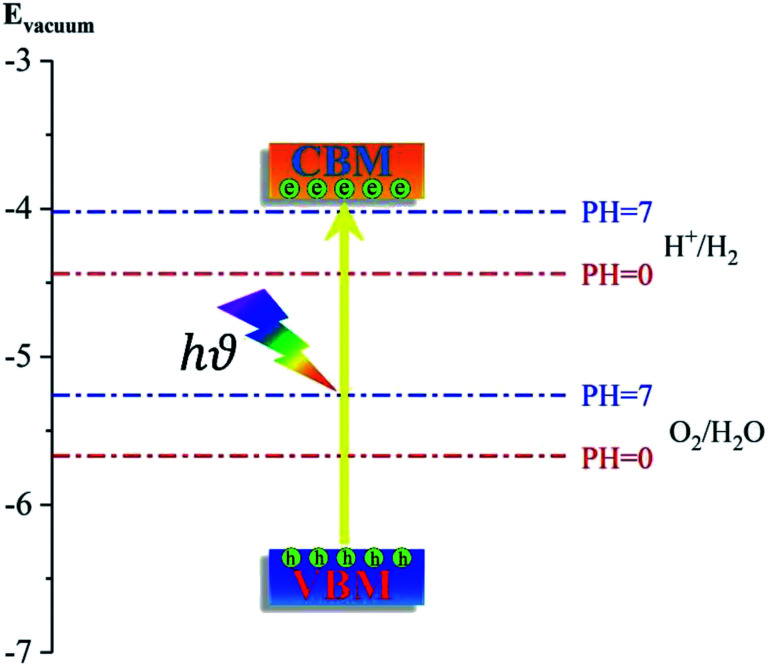
Schematic diagram of the band edge alignment of a penta-Sn_3_H_2_ monolayer sheet with respect to water oxidation (H_2_O/O_2_) and reduction (H^+^/H_2_) potentials. The redox potentials of water splitting at pH = 0 (red dashed lines) and pH = 7 (blue dashed lines) are shown for comparison.

Considering that elemental tin might be unstable in an acidic environment, the redox potential for a water splitting reaction in a neutral environment (pH = 7) was also calculated. The schematic diagram is shown in Fig. 5 for the positions of the band edges of a penta-Sn_3_H_2_ monolayer for photocatalytic water splitting. The position of VBM (−6.49 eV) is lower than the oxygen evolution potential while the position of CBM (−4.05 eV) is higher than the hydrogen evolution potential, fulfilling the thermodynamic requirements for overall water splitting. More importantly, the positions of VBM and CBM can also satisfy photocatalytic water splitting under a highly acidic environment with pH = 0. In addition, the results of PDOS and the charge density distribution of VBM and CBM illustrate that the electrons in CBM are mainly attributed by Sn1 atoms while the holes in VBM are dominated by Sn2 atoms, revealing an excellent electron/hole separation and potential enhancement of the catalytic performance. These results indicate that this material is a candidate for a water-splitting photocatalyst to produce hydrogen without an external bias voltage. More fascinatingly, besides the advantages of suitable positions of band edges in both acidic and neutral environments, a penta-Sn_3_H_2_ monolayer can be modulated into a direct-band-gap semiconductor by strain.

**Fig. 6:**
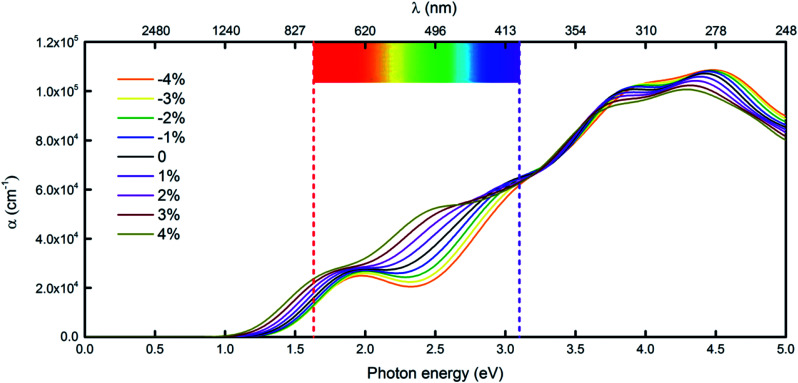
The variation in optical absorption coefficient *α* of penta-Sn_3_H_2_ caused by different tensile strains. The seven-colour-light area between the red and purple lines represents the visible light range.

Another very important condition for photocatalytic water splitting is that the materials should capture a significant fraction of the visible spectrum because visible light with wavelengths of 400–750 nm accounts for 43% of the solar spectrum.^33^ Considering the performance of the penta-Sn_3_H_2_ monolayer under light, we compute its adsorption coefficient.^15,34^ The absorption coefficients with polarization vectors parallel to the layer plane for a penta-Sn_3_H_2_ monolayer are shown in Fig. 6. The decay in light intensity spreading in a unit length of the medium is defined as the absorption coefficient. The penta-Sn_3_H_2_ monolayer exhibits prominent optical absorption in the visible spectrum. The absorption peak also occurred in the infrared light region, indicating a certain amount of infrared light adsorption. More specifically, the absorption coefficient in the blue and UV range is even stronger. Generally, the penta-Sn_3_H_2_ monolayer absorbs considerable light over the entire solar spectrum and adjacent range. Meanwhile, we investigate the effects of biaxial strain on the optical properties of penta-Sn_3_H_2_. The calculated results indicate that the negative strain will enhance the adsorption coefficient while a positive strain decreases it. But in the −4 to 4% range of biaxial tensile strain, the penta-Sn_3_H_2_ still exhibits considerable blue and UV light absorption. The indirect band gap of a penta-Sn_3_H_2_ monolayer can be tuned to a direct one like monolayer technetium dichalcogenides (TcX_2_, X = S, Se),^35^ so they may have potential application in photocatalysts for hydrogen production from water.

**Fig. 7:**
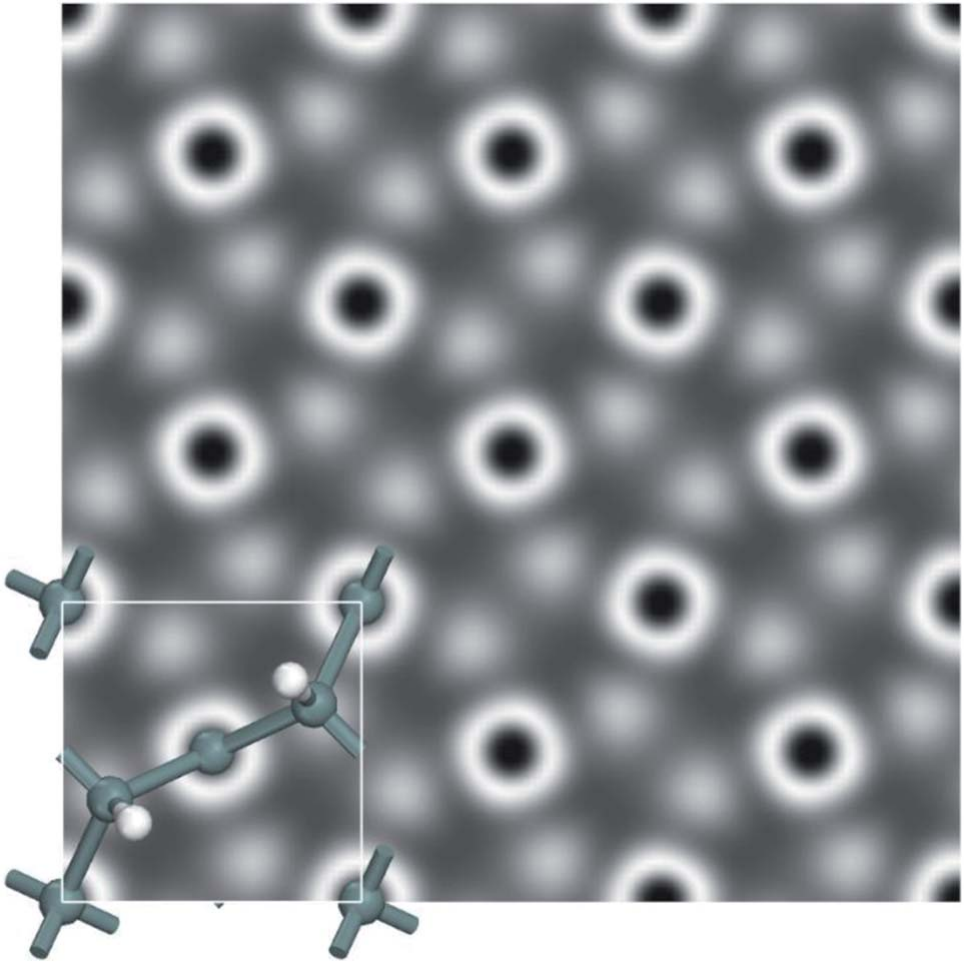
Simulated scanning microscope (STM) images at +2.0 V bias of penta-Sn_3_H_2_.

We further calculated the scanning tunneling microscope (STM) images in order to gain insights into the electronic structure and surface morphology, and also assist future experimental characterization. To help identify these new monolayers in experiments, STM images of a penta-Sn_3_H_2_ monolayer are simulated at +2.0 V bias (Fig. 7). We expect that these features of a penta-Sn_3_H_2_ monolayer will provide more information for identifying this monolayer 2D structure and accelerate the possibility of exfoliating it in the near future.

## 4. Conclusion

In summary, we use first-principles calculations to study the structural stability and electronic properties of pSn modulated by chemical functionalization. Although pristine 2D penta-Sn is energetically unfavorable, penta-Sn_3_H_2_ is found to be dynamically and thermally stable. We have shown that penta-Sn_3_H_2_ is stable even at a high temperature of 900 K. Penta-Sn_3_H_2_ is a semiconductor with an indirect band gap of 1.48 eV (2.44 eV by the HSE06 functional). The electronic structures of the penta-Sn_3_H_2_ can be effectively modulated by biaxial tensile strain. Meanwhile, our calculations reveal that the indirect to direct band gap transition can be achieved for penta-Sn_3_H_2_ sheet by >4% biaxial strain. Most importantly, the penta-Sn_3_H_2_ monolayer absorbs considerable light over the entire solar spectrum and adjacent range, and still exhibits considerable blue and UV light absorption under biaxial tensile strain. Therefore, penta-Sn_3_H_2_ is expected to possess robust structural stability, excellent electronic properties, appropriate band edge position and UV light absorption, allowing Sn-based 2D nanosheets to have potential applications for future nanoelectronics and photocatalytic water splitting. With the development of experimental techniques for the fabrication of 2D materials, we believe that monolayer penta-Sn_3_H_2_ can be achieved in the laboratory in the near future, and its excellent properties and potential applications can be explored. Our work once again highlights that hydrogenation is an efficient approach for modulating the properties of 2D materials, and we hope that our work could stimulate more theoretical and experimental efforts towards designing new materials for nanoelectronics and photocatalysts.

## Conflicts of interest

There are no conflicts to declare.

## Supplementary Material
